# Effect of Internal Defects on the Compression Behavior of Helical Layered Square Honeycombs Fabricated by Selective Laser Melting

**DOI:** 10.3390/ma19122492

**Published:** 2026-06-10

**Authors:** Yue Ni, Yangning Li, Wei Chen, Pengcheng Hu, Xiaobin Li, Wenchao Ke, Jianye Du

**Affiliations:** 1 Ship Shock Damage and Safety Protection Key Discipline and Technology Research Center, Wuhan University of Technology, Wuhan 430063, China; ni@whut.edu.cn (Y.N.); liyangning@whut.edu.cn (Y.L.); whutcw01@126.com (W.C.); lxbmark@163.com (X.L.); douglasdjy@whut.edu.cn (J.D.); 2 Hubei Defense Science and Technology Key Laboratory of Ship Explosion Damage and Protection, Wuhan University of Technology, Wuhan 430063, China; 3 School of Naval Architecture, Ocean and Energy Power Engineering, Wuhan University of Technology, Wuhan 430063, China; 4 Hubei Digital Manufacturing Key Laboratory, School of Mechanical and Electronic Engineering, Wuhan University of Technology, Wuhan 430070, China; wenchaoke@whut.edu.cn

**Keywords:** selective laser melting, manufacturing defects, in-situ CT, GTN damage model

## Abstract

The emergence of selective laser melting (SLM) has enabled the fabrication of complex structures with exceptional mechanical performance. However, process-induced defects, including porosity and geometric deviations, pose significant challenges to structural reliability, and their dynamic evolution under loading remains poorly understood. In this study, helical layered square honeycomb structures were fabricated via SLM. The effects of process conditions on defect characteristics, as well as the influence of porosity and wall thickness defects on mechanical properties, were investigated using X-ray computed tomography (CT), in situ loading tests, and finite element simulation. The results indicate that the investigated high-quality process conditions minimize porosity, optimize pore morphology, and improve wall thickness uniformity, thereby substantially reducing the adverse effects of pores on tensile properties. Under compressive loading, defect evolution, including pore expansion and wall thickness thinning, is primarily concentrated at structural corners, with more pronounced variations observed under coarse process conditions. Increased porosity, wall thickness reduction, and uneven thickness distribution all degrade the quasi-static compressive performance and medium to high-velocity impact resistance of the structure. Furthermore, thickness distribution exerts an independent influence on mechanical properties beyond the effect of overall average thickness.

## 1. Introduction

The advent of additive manufacturing (AM), particularly Selective Laser Melting (SLM), has revolutionized the design and fabrication of complex metallic structures. In the field of protective engineering, this technology unlocks unprecedented potential for developing bio-inspired lightweight meta-structures. By mimicking sophisticated architectures found in nature, such as lattice and cellular designs, these structures promise exceptional specific strength, tunable energy absorption, and multi-functional performance. For instance, triply periodic minimal surface structures and functionally graded lattices have been demonstrated to possess superior energy absorption capabilities and designable mechanical properties [1,2,3]. More recently, bio-inspired glass-sponge lattices, chiral energy-absorbing structures, and helical layered honeycombs have further demonstrated that architectural topology can strongly affect compressive strength, deformation recovery, and energy absorption efficiency [4,5,6]. These attributes are key requirements for next-generation protective applications in aerospace, automotive, and defense sectors. The ability of SLM to fabricate intricate, near-net-shape geometries directly from digital models eliminates many constraints of traditional manufacturing, enabling the realization of highly optimized, topologically complex designs that were previously impractical or impossible to produce [7,8].

However, the transition of these advanced designs from concept to reliable applications is critically hampered by the inherent defect-sensitive nature of the SLM process. The rapid, layer-wise melting and solidification dynamics, coupled with complex thermal histories, inevitably introduce internal flaws [9,10,11,12,13]. Hojjatzadeh et al. and Khairallah et al. [14,15] directly observed the dynamic formation mechanisms of gas pores and lack-of-fusion pores during the laser powder bed fusion process using high-speed imaging techniques. Complementing these findings, Guo et al. [16] employed high-speed high-energy X-ray imaging to reveal the transient dynamics of powder spattering and its correlation with defect generation, while Zhao et al. [17] demonstrated real-time monitoring capabilities for melt pool dynamics and defect formation. An et al. [18] conducted a quantitative study on the geometric characteristics and formation mechanisms of porosity defects in Ti6Al4V alloy using micro-computed tomography (μ-CT).

It should be noted that these imperfections are not identical in origin or mechanical effect. Gas pores and keyhole pores are mainly associated with melt-pool instability and vapor depression, whereas lack-of-fusion defects are generally related to insufficient energy input or incomplete track overlap. In contrast, geometric deviations and surface roughness are more closely associated with scanning strategy, powder adhesion, thermal distortion, and the limited forming resolution of SLM. Moreover, geometric deviations from the intended design, including strut thickness variations and surface roughness induced by scanning strategies, are frequently encountered [19,20]. For thin-walled lattice structures that constitute bio-inspired meta-structures, these manufacturing imperfections are not merely incidental; they act as intrinsic stress concentrators and preferential sites for damage initiation. Consequently, they can precipitate catastrophic reductions in mechanical performance. Substantial research indicates that porosity and geometric defects significantly degrade material fatigue strength, exacerbate performance scatter, and alter fatigue crack growth behavior [21,22,23,24,25,26]. In addition, recent studies on additively manufactured alloys, including high-entropy alloys, have emphasized that defect behavior is often coupled with microstructural features such as grain morphology, texture, residual stress, and phase constitution, highlighting the importance of considering the processing–structure–defect–property relationship [27]. This not only undermines the predicted performance and structural integrity but also casts significant uncertainty on the reliability of AM-fabricated protective components.

Current approaches to assessing AM part quality often operate at disparate scales. Non-destructive evaluation techniques like μ-CT provide excellent volumetric quantification of defect populations in the as-built state and have been widely applied for quality control of various alloys [28,29,30]. Meanwhile, macroscopic mechanical testing evaluates the final performance outcome. A significant knowledge gap exists in dynamically linking the in situ evolution of these micro/meso-scale defects under load to the ultimate macroscopic failure. More specifically, it remains unclear how process-induced porosity and geometry deviations jointly affect damage initiation, collapse localization, and energy absorption in defect-sensitive bio-inspired honeycomb structures. Conventional constitutive models often treat AM materials as fully dense, isotropic continua, failing to capture the progressive damage nucleation and coalescence initiated at defect sites [21,22]. Jiang et al. [31] explicitly highlighted that defects significantly influence the elastic response of lattice structures, underscoring the limitations of models that neglect defect characteristics. Although continuum damage mechanics models, such as the Gurson–Tvergaard–Needleman (GTN) model [32,33,34,35], offer a framework for simulating ductile fracture via pore evolution, their accurate application to SLM materials requires precise calibration of damage parameters that reflect specific process-induced microstructure and defect characteristics [36]. As demonstrated by the work of He et al. and Abbassi et al. [37,38], the determination of GTN model parameters often relies on sophisticated in situ experiments or inverse optimization algorithms. A generalized, one-size-fits-all parameter set lacks predictive fidelity for materials produced under varying process conditions.

To bridge this multi-scale gap, a comprehensive methodology that integrates advanced characterization, in situ experimentation, and calibrated predictive modeling is essential. This study establishes an integrated framework to elucidate the effect of intrinsic manufacturing defects on the mechanical behavior of SLM-fabricated 316L stainless-steel bio-inspired protective structures. The work is systematically pursued through three main thrusts. First, statistical quantification of defect populations is performed using μ-CT. In SLM-fabricated 316L stainless steel, pores and wall-thickness deviations may coexist with residual stress, unmelted particles, grain texture, and melt-pool boundaries. Therefore, the measured defects should be interpreted as geometric manifestations of process instability rather than isolated geometrical imperfections. Second, tracking damage evolution is performed via in situ mechanical loading coupled with interval CT scanning. Lastly, inverse calibration of a GTN damage model is performed using experimental data. This aims to establish a validated predictive modeling framework. This study provides an experimentally supported correlation among representative process conditions, CT-measured defects, in situ damage evolution, and macroscopic mechanical response. The results offer useful insights for the defect-tolerant design and performance evaluation of load-bearing additively manufactured metallic lattice structures. This work provides a quantitative reference for predicting the mechanical performance of such structures under realistic manufacturing conditions.

## 2. Materials and Methods

### 2.1. Materials and Sample Preparation

**Figure 1:**
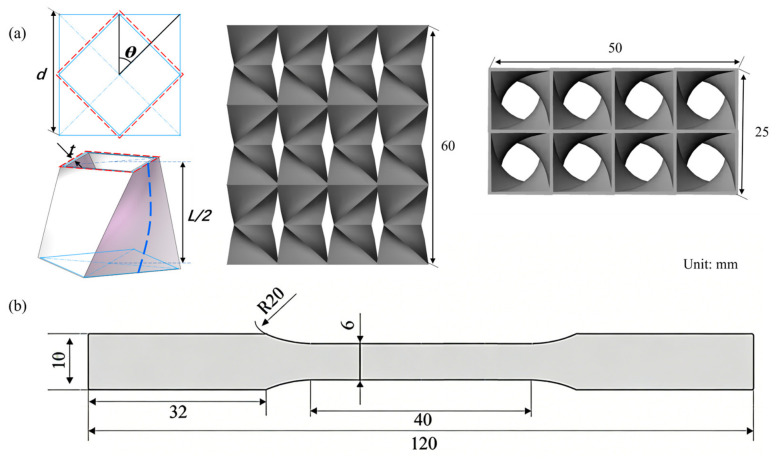
(**a**) The geometry of HLSHS; (**b**) dimensions of tensile specimen.

Helical layered square honeycomb structure (HLSHS) refers to a square honeycomb topology with layer-wise helical rotation (Figure 1a). The design in this study combines the characteristics of a conventional square honeycomb and a helical configuration. As illustrated in Figure 1a, the unit cell of the proposed structure has a helix angle θ = 90°, a wall thickness t = 0.5 mm, a width d = 12.5 mm, and a length L = 20 mm. The overall structure consists of unit cells arranged in a 4 × 3 × 2 array, with final overall dimensions of 60 mm × 50 mm × 25 mm. Corresponding tensile specimens were synchronously fabricated for this structure, and the specimen dimensions are detailed in Figure 1b.

**Figure 2:**
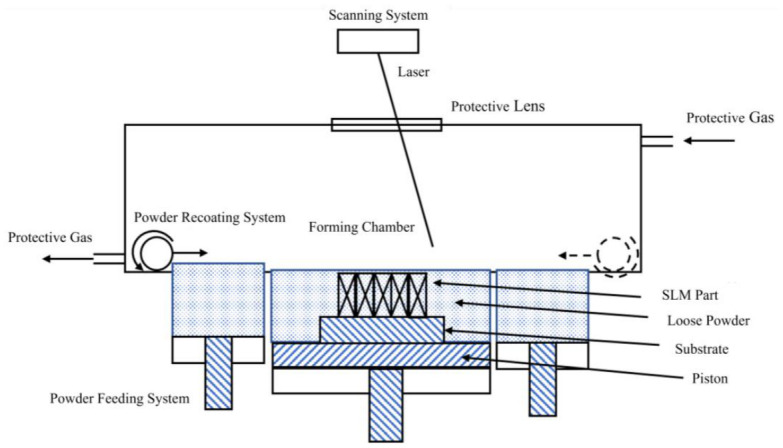
The Schematic of the SLM280 Equipment.

The material used for machining in this study was gas-atomized 316L stainless-steel powder with a particle size of 15–53 μm. 316L stainless steel exhibits excellent biocompatibility, corrosion resistance, and mechanical properties. The SLM280 system (Nikon SLM Solutions AG, Lübeck, Germany) (Figure 2) was selected for this study, employing a bidirectional powder spreading method with a squeegee. Nitrogen and argon gases were introduced during processing to shield the system, supported by an efficient protective gas recirculation system to maintain a clean processing environment.

To investigate the 3D printing process parameters, nine 1 cm × 1 cm × 1 cm 316L metal cube specimens were experimentally prepared using different process parameters. By comparing the density of the specimens, suitable 3D printing process parameters were selected. Density is a key criterion for assessing the quality of additive manufacturing specimens. It is defined as the ratio of the specimen density after additive manufacturing to the standard density of 316L stainless steel (expressed as a percentage), reflecting the internal porosity of the specimen. After cleaning and drying the 316L stainless-steel specimens produced by additive manufacturing, their density was measured using the Archimedes displacement method. The density under different processing parameters is shown in Table 1. The Archimedes displacement method was selected because it is a widely used, simple, and non-destructive method for evaluating the relative density of additively manufactured metallic parts. In this study, it was used as a preliminary screening tool to compare the forming quality of specimens fabricated using different SLM processing parameters. However, this method provides only an overall density value and cannot distinguish closed pores, lack-of-fusion defects, or microcracks. Therefore, X-ray CT characterization was further performed to quantify pore size, morphology, and spatial distribution.

**Figure 3:**
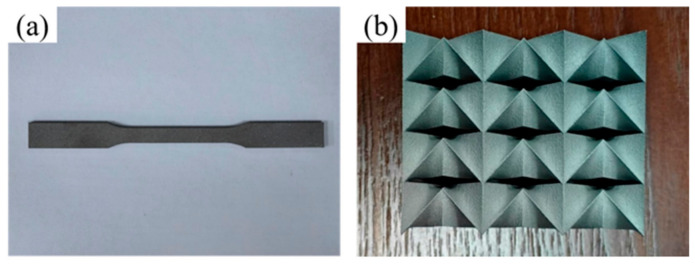
Dimensions of the additive manufacturing model and physical object: (**a**) tensile test specimen, (**b**) HLSHS.

This study selects two sets of selective laser melting (SLM) processing parameters with distinct forming quality (one optimal and one defective) to investigate the influence of process-induced defects on the mechanical performance of HLSHS. The two parameter sets are defined as follows: ① High-quality (HQ) group (original T8): Laser power 260 W, scanning speed 1200 mm/s, scan pitch 0.15 mm, powder layer thickness 0.04 mm; ② Low-quality (LQ) group (original T7): Laser power 235 W, scanning speed 1000 mm/s, scan pitch 0.15 mm, powder layer thickness 0.04 mm. These two conditions were not intended to represent the complete process space but were selected as representative high- and low-quality cases for defect-mechanics comparisons. The quasi-static tensile test specimens and the HLSHS were fabricated using 316L metal powder via an SLM280 additive manufacturing machine. The physical SLM-processed specimens are shown in Figure 3.

### 2.2. Morphology Characterization

In this study, the YXLON FF35 (YXLON International GmbH, Hamburg, Germany) micro-focus CT system was employed to scan and examine the internal pores within the tensile specimens and the HLSHS. By performing CT scanning on the gauge section of the 316L stainless-steel tensile specimen, fine pores distributed throughout the specimen were captured. These pores exhibited a diffuse distribution within the specimen, reflecting the sensitivity and capability of CT scanning to detect internal density variations in the material. To quantitatively characterize the geometric defects and their spatial distribution for quality inspection and finite element modeling, XCT scanning was further conducted on the HLSHS.

Porosity is used in this study as a measurable descriptor of process-induced internal defects, while its formation may originate from melt-pool instability, insufficient fusion, gas entrapment, or local thermal history. The size and sphericity of pore defects are the most critical parameters characterizing pore size and morphology, exerting the greatest influence on the mechanical properties of tensile specimens produced via additive manufacturing. In addition to size and sphericity, pore clustering, aspect ratio, connectivity, and distance to stress-concentration regions may also affect crack initiation. These descriptors will be included in future quantitative defect–property models [39].

Sphericity *ψ* describes the degree to which a pore defect approaches a perfect sphere, calculated using the following expression. A value closer to 1 indicates a more spherical shape.

(1) $$\psi =\frac{\sqrt[{3}]{36\pi V^{2}}}{S}$$

Here, *V* is the pore volume and *S* is the pore surface area.

### 2.3. In-Situ Loading Test

In this study, in situ tensile tests were conducted at room temperature using a universal testing machine (CMT4104 (MTS Systems (China) Co., Ltd., Shenzhen, China)). Tensile tests were performed on two tensile specimens fabricated by different processing techniques to monitor the deformation evolution and internal pore changes in the gauge section during loading. An interrupted in-situ CT scanning strategy was adopted: the specimen was first loaded to a preset displacement or load value and held constant, after which the gauge section at that state was scanned using the YXLON FF35(YXLON International GmbH, Hamburg, Germany)micro-focus CT system. The internal defects of the SLM-fabricated helical layered square honeycomb structures were characterized using a METROTOM 1500 micro-CT system(Carl Zeiss Industrielle Messtechnik GmbH, Oberkochen, Germany). The scanning parameters were set as follows: spatial resolution of 60 lp/mm, X-ray source focal spot size of 4 μm, and a 2000 × 2000 area-array detector. This “load-hold-scan” process was repeated until specimen failure. The interrupted CT strategy may introduce stress relaxation during the holding period. Therefore, the reconstructed defect evolution should be interpreted as the evolution under quasi-static interrupted loading rather than continuous loading. To ensure consistent image resolution across different scanning states, the scanning field of view was kept unchanged throughout the in situ loading process; consequently, during tensile deformation, portions of the upper and lower ends of the gauge section moved out of the field of view. The tensile loading rate was 0.1 mm/min, and the maximum test force capacity of the testing machine was 100 kN.

**Figure 4:**
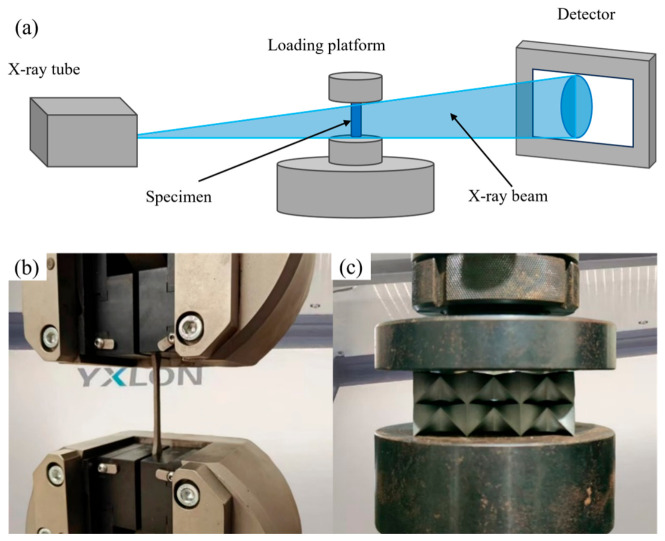
In situ tensile test: (**a**) In situ loading schematic diagram, (**b**) tensile test, (**c**) compression test.

In situ compression tests were conducted using a universal testing machine (LD26.10 (Lishi (Shanghai) Instrument Co., Ltd., Shanghai, China)), employing the same interrupted in situ scanning strategy as the tensile tests. The compression loading rate was 0.5 mm/min, and the maximum test force capacity was 100 kN. The in situ loading test setup is shown in Figure 4.

### 2.4. Numerical Simulation

**Figure 5:**
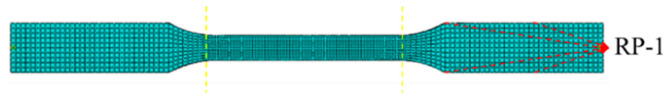
Tensile simulation model.

A finite element model of the tensile specimen matching the actual test dimensions was established in Abaqus/Explicit 2021. The finite element model and constraints are shown in Figure 5. The C3D8R eight-node linear hexahedral element was selected for the tensile structure mesh. To ensure computational accuracy, the mesh was refined in the central region of the specimen, with a mesh size of 0.5 mm. The mesh size at both ends of the specimen was set to 1 mm, with a transition mesh between the two types. The total number of elements for the entire tensile specimen was 6400. When setting the load, we fully clamped the left end of the specimen and coupled the right end to the reference point to apply a displacement load simulating tensile testing. After completing the calculation, we output the force–displacement data and convert it into an engineering stress–strain curve to establish a response surface model.

**Figure 6:**
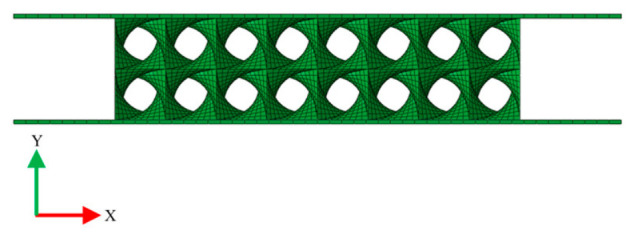
Finite element model of a HLSHS.

To investigate the influence of pore defects on the mechanical properties of HLSHS, a finite element model was established and analyzed, as shown in Figure 6. The helical layered square honeycomb structures model was positioned between two plates. The top plate was defined as a discrete rigid body, while the bottom plate was fixed. The top plate impacted the bottom plate at a constant velocity of 1 m/s. General contact with a friction coefficient of 0.2 was applied to the structure itself and its contact with both plates. To eliminate boundary effects during compression and ensure result convergence, the plate-frame honeycomb was meshed with 8, 2, and 5 cells in the X, Y, and Z directions, respectively. Mesh generation employed 1.2 mm shell elements, totaling 51,200 elements. The matrix material utilized 316L stainless steel, with material parameters shown in Figure 18. The material was assumed to be isotropic in the finite element model. This simplification neglects SLM-induced anisotropy associated with grain texture and melt-pool boundaries. The GTN model was incorporated as the damage model in the finite element analysis, with model parameters configured as listed in Table 6. Since a strain-rate-dependent constitutive model was not explicitly included, the impact simulations are used to compare the relative effects of different defect levels under consistent assumptions. Quantitative prediction of high-rate impact response requires future calibration of rate-dependent parameters for SLM 316L stainless steel.

Tvergaard and Needleman [35,40] revised the Gurson model by introducing three correction factors—*q*_1_, *q*_2_, and *q*_3_—to refine the yield function within the original model. While the original model only considered monotonically increasing porosity, the modified GTN numerical model decomposes damage evolution into three stages: nucleation, growth, and coalescence. This effectively simulates the progressive damage evolution of internal pores within materials during tensile loading. The revised GTN model expression is as follows [31]:

(2) $$\phi ={\left(\frac{\sigma_{e}}{\sigma_{y}}\right)}^{2}+2q_{1}f^{*}\left(\frac{3q_{2}\sigma_{m}}{2\sigma_{y}}\right)-1-q_{3}{\left(f^{*}\right)}^{2}=0$$

where f* is the effective porosity, used to correct the original porosity and describe the progressive damage evolution during the stage of pore coalescence. When the pore volume fraction *f* within the material reaches a critical value, f* accelerates its growth, reflecting the process of pores merging into macroscopic cracks. *σ_m_* denotes hydrostatic stress, reflecting the mechanical state of volumetric deformation in the material and playing a crucial role in pore growth.

When setting up the GTN damage numerical model for simulation calculations, nine parameters must be determined: *f*_0_, *f_n_*, *f_c_*, *f_f_*, *q*_1_, *q*_2_, *q*_3_, *S_N_*, and *ε_N_*. The final values adopted are *q*_1_ = 1.5, *q*_2_ = 1.0, *q*_3_ = 2.25 [34]. *S_N_* and *ε_N_* are often treated as constants in studies. *ε_N_* represents the average nucleation equivalent plastic strain, while *S_N_* denotes the nucleation strain standard deviation. Based on references, these two parameters were set to *S_N_* = 0.1 and *ε_N_* = 0.1 [41].

To investigate the microstructure evolution and damage mechanism during the tensile process, in situ observation of the specimen microstructure at different deformation stages was conducted using a field emission scanning electron microscope (FE-SEM). Quantitative analysis of the SEM images was performed using ImageJ (Version 1.54r) image processing software. By measuring the pore area in the images, the internal pore volume fraction of the specimen at different tensile stages was calculated. Multiple measurements were taken for each image and averaged to reduce measurement errors introduced by the software, and the GTN damage parameters were ultimately obtained. Establishing a response surface model is primarily determined by two components: response factors and response values. For the GTN model response surface construction, this paper treats the maximum stress point and fracture point directly obtained from the stress–strain curve in tensile testing as characteristic points of the stress–strain curve. The four key values at these characteristic points—maximum strain R_1_, maximum stress R_2_, fracture strain R_3_, and fracture stress R_4_—serve as response values. The four parameters *f*_0_, *f_n_*, *f_c_*, and *f_f_* of the GTN model are treated as influencing factors. Using Design Expert and a central composite design method, 28 uniaxial tensile calculation scenarios were designed, as shown in Table 2.

## 3. Results and Analysis

### 3.1. Defect Statistical Characteristics Analysis

#### 3.1.1. Tensile Test Specimen

**Figure 7:**
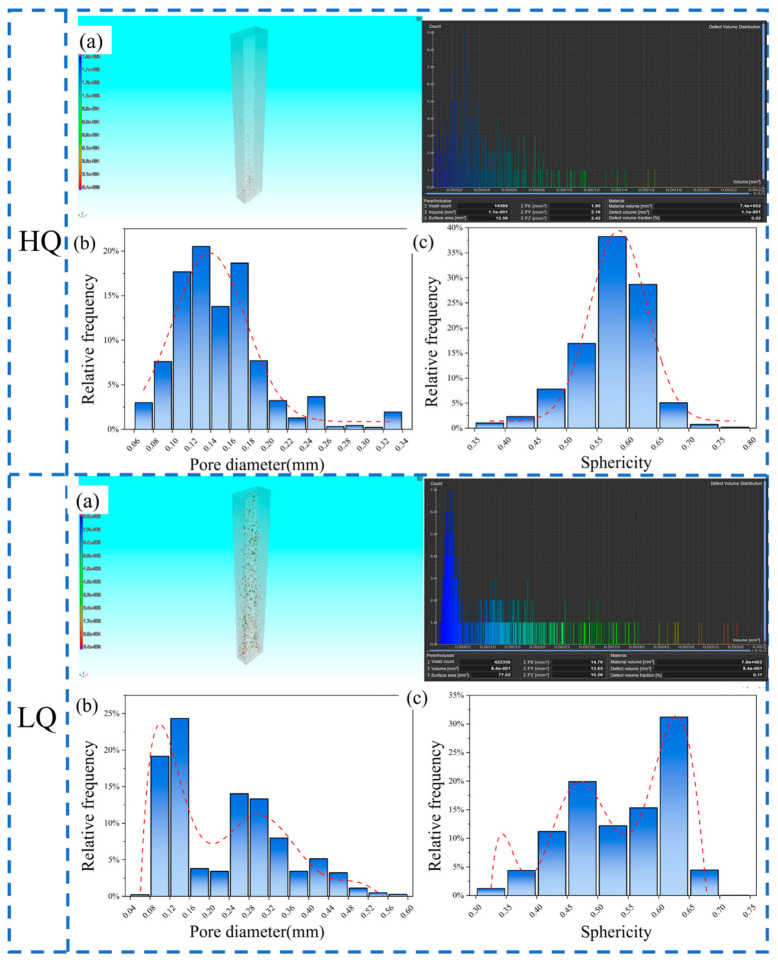
(**a**) CT scan, (**b**) probability distribution plot and distribution curve fitting for diameters of pore defects, (**c**) probability distribution plot and distribution curve fitting for sphericity of pore defects.

CT scans (Figure 7a) clearly capture density variations within the specimens, revealing uniformly distributed spherical micropores throughout the interior with no detectable inclusions or other defects. The HQ tensile specimen exhibited a pore volume fraction of 0.02%, while the LQ specimen showed a pore volume fraction of 0.11%.

The pore diameter distributions of the two specimens exhibit significant differences. The pore diameters of the HQ specimen are concentrated in the range of 0.06–0.34 mm, presenting a left-skewed distribution, indicating that small pores predominate. The main peak lies in the 0.12–0.14 mm interval, with a relative frequency of 20.51%, while a secondary peak appears in the 0.16–0.18 mm interval (18.64%). Beyond 0.18 mm, the relative frequency rapidly decreases to below 10%, suggesting that large pores are relatively rare in this specimen. In contrast, the LQ specimen exhibits a wider pore diameter distribution range of 0.04–0.60 mm, with an overall bimodal distribution. The main peak occurs in the 0.12–0.16 mm interval, with a relative frequency of 24.34%; however, the frequency drops sharply to below 5% for diameters exceeding 0.16 mm. A secondary peak is observed in the 0.24–0.28 mm interval, with a relative frequency of 14.03%. Compared with the HQ specimen, the LQ specimen not only contains more small pores but also has a certain proportion of larger pores (>0.24 mm), reflecting a more dispersed pore size distribution.

The pore sphericity distributions of the two specimens also show marked differences. The sphericity of pores in the HQ specimen is concentrated in the range of 0.35–0.80, exhibiting a pronounced concentrated distribution. The main peak lies in the 0.55–0.60 interval, accounting for 38.24% of the total, and the cumulative proportion in the 0.50–0.65 interval reaches 83.83%, with extremely few pores outside this range. This indicates that the vast majority of pores in the HQ specimen are relatively regular in shape and exhibit high sphericity. The sphericity of pores in the LQ specimen ranges from 0.30 to 0.75, with an overall sphericity level lower than that of the HQ specimen and a more dispersed distribution. The main peak lies in the 0.60–0.65 interval, accounting for 31.20%, while pores with sphericity below 0.5 account for 36.73%, significantly higher than that of the HQ specimen. This suggests that the LQ specimen contains many irregularly shaped pores, indicating poorer uniformity in pore morphology. In summary, the HQ specimen is characterized by concentrated pore sizes and regular pore shapes, whereas the LQ specimen exhibits a wider size distribution and more irregular pore shapes, reflecting the significant influence of the two processing techniques on pore structure.

#### 3.1.2. HLSHS

**Figure 8:**
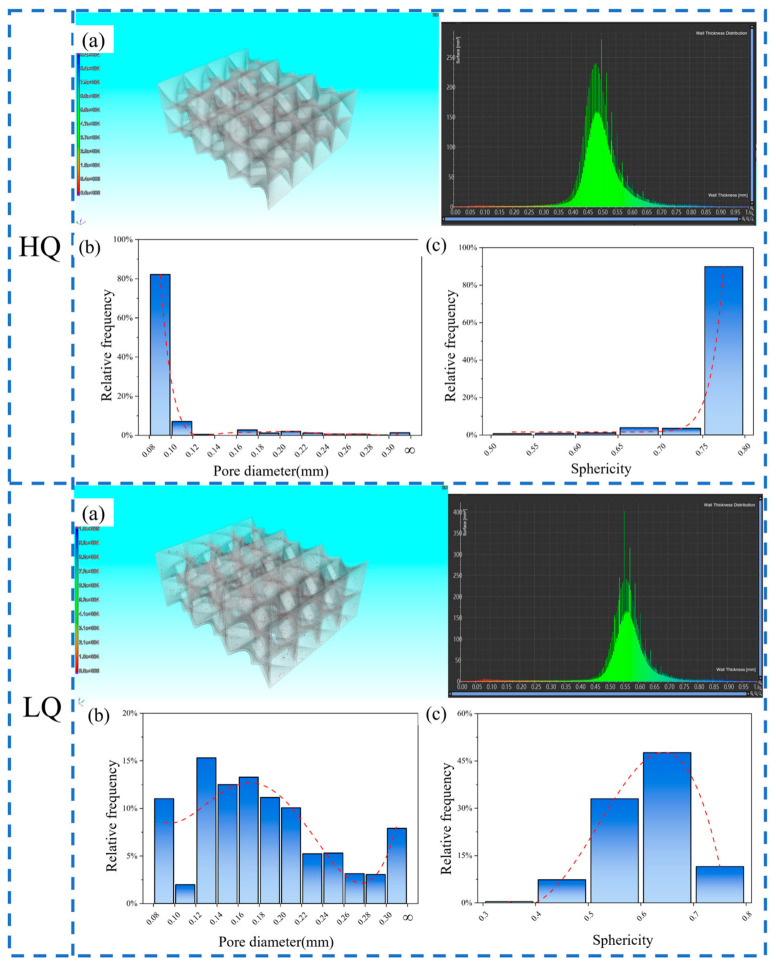
(**a**) CT scan, (**b**) probability distribution plot and distribution curve fitting for diameters of pore defects, (**c**) probability distribution plot and distribution curve fitting for sphericity of pore defects.

CT scan (Figure 8a) results reveal that the fine pores within the HLSHS exhibit a regular distribution pattern, primarily concentrated at structural corners and high-twist regions—corresponding to the process weak points in additive manufacturing. The pores are predominantly spherical in shape, with no inclusions or other defects detected. The porosity volume fraction for the HQ specimen is 0.03%, while that for the LQ specimen is 0.08%.

Statistical analysis of pore diameter distribution (Figure 8b) indicates that pores in the HQ specimen predominantly measure below 0.1 mm, accounting for 90.66% of the total. The LQ specimen exhibits a broader pore diameter range with significantly larger overall dimensions compared to HQ, where pores below 0.2 mm constitute only 54.13% of the total.

The statistical results of pore sphericity distribution (Figure 8c) show that the pore sphericity distribution range for HQ specimens was 0.50–0.80, with the 0.75–0.80 range accounting for as much as 89.88%, indicating high regularity of pore shape. For LQ specimens, the pore sphericity distribution ranged from 0.30 to 0.80, with a notable increase in irregular pores. Sphericity was concentrated in the 0.50–0.70 range, accounting for 80.68%.

### 3.2. Defect Evolution Behavior Under In Situ Loading

#### 3.2.1. Tensile Test Specimen

**Figure 9:**
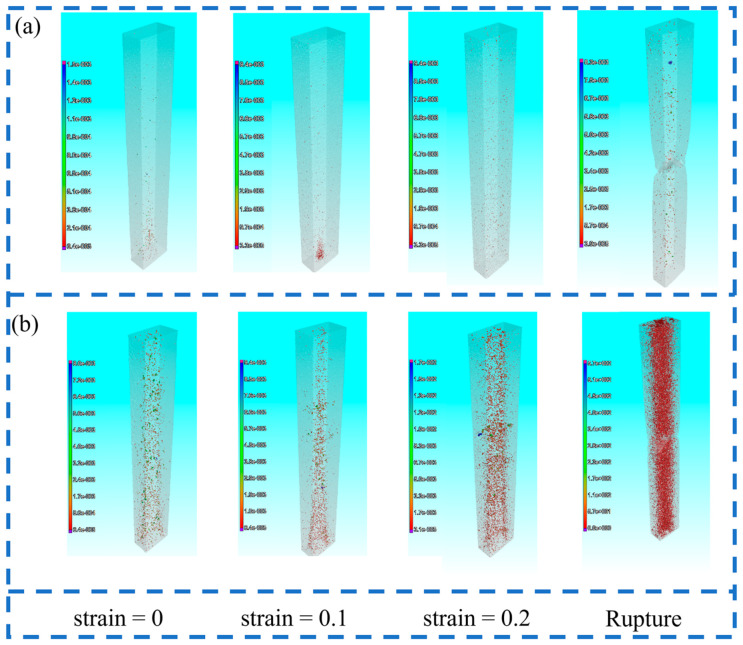
In situ loading process of tensile specimens: (**a**) HQ, (**b**) LQ.

The tensile specimen prepared using the HQ (Figure 9a) method exhibited an initial porosity of 0.02%, with pores primarily concentrated beneath the tensile section. At a tensile strain of 0.1, the specimen showed no significant deformation, with only a slight increase in pore count, most notably in the original pore-concentrated zone. At a strain of 0.2, the specimen exhibited slight necking, accompanied by a further increase in pore count and enlarged pore diameters in some pores. During the fracture process, both pore count and pore size increased substantially, with some pores coalescing into larger pores. The observed pore population showed limited influence on the tensile response under the present testing conditions. The regions of high pore concentration and large pore formation did not coincide with structural damage locations. Thus, under optimal fabrication conditions, the impact of pores on structural mechanical properties is limited.

The tensile specimen prepared from LQ (Figure 9b) had an initial porosity of 0.11%, with pores uniformly distributed throughout the tensile section. At a tensile strain of 0.1, the specimen showed no significant deformation, but both the number and diameter of pores increased markedly. At a strain of 0.2, slight necking occurred, with the number of pores further increasing. Within the necking zone, some pores coalesced to form numerous large pores. During the fracture process, the number of pores increased dramatically, covering almost the entire tensile section. After the fracture, the original large pores disappeared. Throughout the tensile process, pores significantly influenced the structural deformation behavior. The locations where large pores formed highly coincided with structural damage sites, and the pore aggregation zones served as fracture initiation points. Therefore, under rough processing conditions, pores exerted a significant negative impact on the structural mechanical properties.

**Figure 10:**
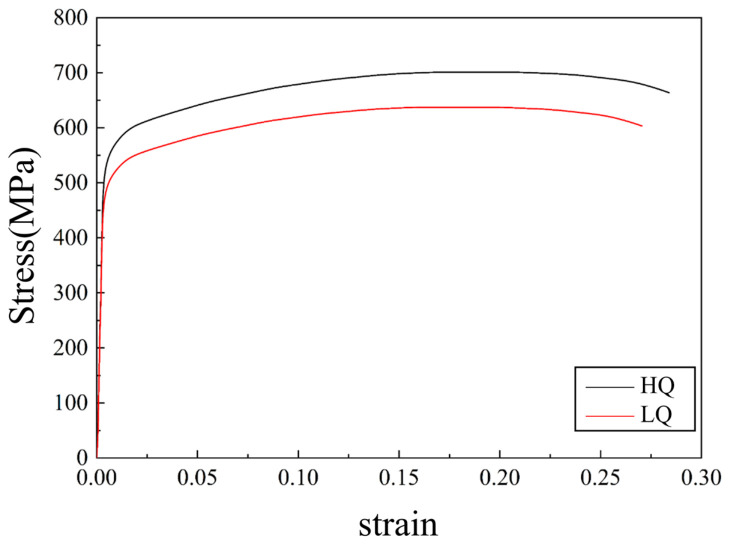
In situ tensile test stress–strain curve.

The stress–strain curve from the in situ tensile test (Figure 10) indicates that the LQ specimen, with its higher initial porosity and significant influence of pore evolution on its mechanical properties, exhibits both lower yield strength and tensile strength compared to the HQ specimen. Additionally, due to the high correlation between pore aggregation locations and fracture initiation points, its fracture strain also occurs significantly earlier.

#### 3.2.2. HLSHS

The HLSHS prepared by HQ exhibits an initial porosity of 0.03%, with pores primarily concentrated at structural corners and regions of abrupt angular changes. The LQ specimen shows an initial porosity of 0.08%, with pores widely distributed throughout the entire structure.

**Figure 11:**
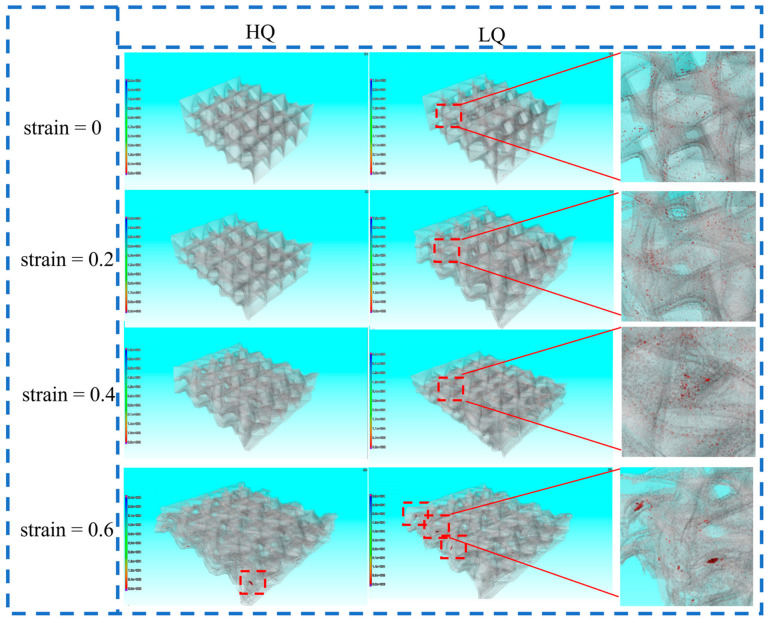
Pore development process.

The evolution of pores during compression is as follows (Figure 11): At a strain of 0.2, the HQ specimen undergoes folding deformation due to its helical structure design, resulting in a slight increase in pore count, with a modest expansion in pore volume and diameter within the deformed zone. In the LQ specimen, both pore count and diameter significantly increase in the deformed zone, with adjacent pores showing a tendency to coalesce. At 0.4 strain, pore count in the HQ specimen further increased, with distribution expanding throughout the entire structure; pores in the LQ specimen continued to grow significantly, and the aggregation trend intensified. At 0.6 strain, pore aggregation occurred in both specimen groups within large deformation regions such as corners, forming multiple large pores. The concentration of defects at corners may result from the combined effects of local heat accumulation during SLM, scan-path transition, geometric complexity, and stress concentration during loading.

**Figure 12:**
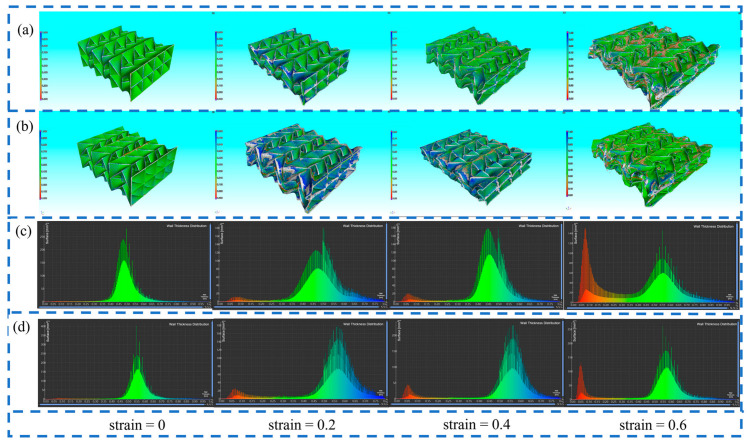
Wall thickness variation process. (**a**) XCT scan of wall thickness (HQ); (**b**) Wall thickness histogram (HQ); (**c**) XCT scan of wall thickness (LQ); (**d**) Wall thickness histogram (LQ).

Regarding wall thickness (Figure 12), the initial average wall thickness of the HQ specimen was 0.502 mm (nominal thickness 0.5 mm), closely matching the nominal value with uniform thickness distribution. The initial average wall thickness of the LQ specimen was 0.568 mm (nominal thickness 0.5 mm), showing significant deviation from the nominal value with uneven thickness distribution, where the initial wall thickness at corners was notably lower than in other regions. During compression, the structural walls deformed under combined tensile and compressive stresses, causing wall thickness to increase or decrease. Both specimen groups exhibited consistent thickness evolution patterns: at 0.2 strain, wall thinning occurred at corners while spiral surfaces thickened; at 0.4 strain, overall thinning progressed; at 0.6 strain, structural collapse occurred with multiple wall sections reducing to 0 mm thickness. Specimen LQ exhibited more pronounced wall thickness distribution irregularities throughout the test due to insufficient initial forming precision.

**Figure 13:**
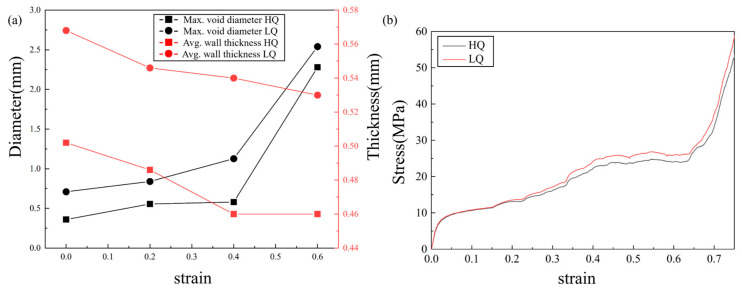
(**a**) Defect parameters of HLSHS under different strains, (**b**) in situ compression test stress–strain curve.

The stress–strain curves from the in situ compression tests (Figure 13b) reveal similar deformation behaviors for both specimen groups, with largely consistent curve trajectories. During the elastic stage and early plastic deformation, the two curves closely overlap, indicating that differences in wall thickness and porosity did not significantly affect mechanical properties at this stage. As strain continued to increase, the LQ specimen exhibited a transient decrease in stress due to its greater formed thickness, higher stress levels, and increased porosity. The failure of HLSHS should be interpreted as a coupled multi-scale process. Microscale pore growth contributes to local material degradation, while mesoscale wall folding, corner deformation, wall-thickness thinning, and macroscale structural collapse dominate the overall compressive response. In this study, the GTN model was used as an effective continuum damage model to describe ductile damage in the 316L matrix and to compare the relative influence of different defect levels. This approach is reasonable when pores are relatively dispersed and their characteristic size is smaller than the local wall thickness. However, homogenization may break down when pores become strongly clustered or connected, when pore size becomes comparable to the wall thickness, or when a localized crack path forms across thin walls or corner regions. Under such conditions, discrete fracture, cohesive-zone, phase-field, or percolation-based models may be more suitable. Future work will further consider these approaches to describe highly localized defect interaction and crack propagation in SLM-fabricated HLSHS.

Overall, both pore distribution and wall thickness undergo significant evolution with increasing compressive strain: Pore count and diameter continuously increase with strain, with the maximum pore diameter in LQ specimens growing from 0.71 mm to 2.54 mm, and in HQ specimens from 0.36 mm to 2.28 mm. Wall thickness exhibited an overall thinning trend, with the average wall thickness of the LQ specimen decreasing from an initial 0.568 mm to 0.53 mm, and that of the HQ specimen decreasing from 0.502 mm to 0.46 mm.

### 3.3. Damage Parameter Optimization and Model Validation

#### 3.3.1. Determination of GTN Parameter Range

**Figure 14:**
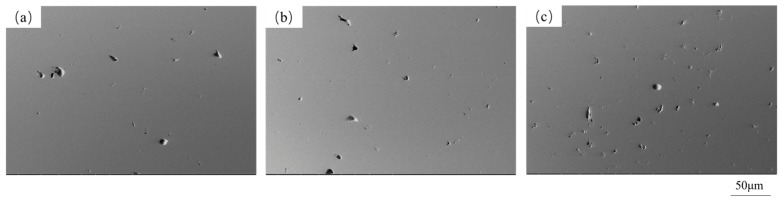
SEM images at different deformation stages: (**a**) before deformation; (**b**) necking; (**c**) fracture.

In situ observation of the microstructure of specimens at different deformation stages yielded the results shown in Figure 14. The figure reveals that as deformation progresses, the volume of existing pores increases while new pores nucleate, leading to a rise in pore count. The sheet exhibits a small number of minute pores in its initial state, which is attributed to the SLM manufacturing process. Most metal structures inherently contain some internal porosity defects after fabrication.

Quantitative analysis of the SEM images was performed using ImageJ image processing software. By measuring the pore volumes in the photographs, the internal pore volume fractions at different tensile stages of the specimens were obtained. Each photograph required multiple measurements to calculate the average value, eliminating software measurement errors. The final GTN damage parameters are shown in Table 3.

**Figure 15:**
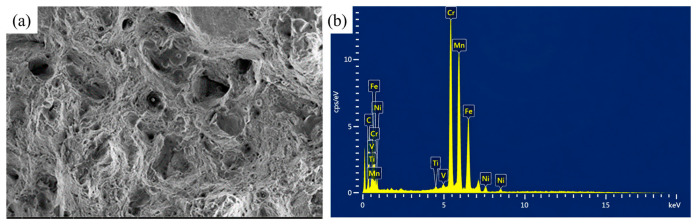
EDS measurement results: (**a**) Scanned cross-section, (**b**) elemental energy spectrum.

In the cross-section shown in Figure 15a, pores are observed to nucleate preferentially at second-phase particles or inclusions. Energy-dispersive spectroscopy (EDS) was performed on the tensile fracture surface of the 316L stainless-steel specimen to analyze the composition of the second phase. The corresponding EDS spectrum is presented in Figure 15b, where the horizontal axis represents energy (keV) and the vertical axis denotes count intensity (cps/keV). The figure indicates the sample primarily contains elements such as Fe, Cr, Mn, and Ni. Fe serves as the matrix element with prominent peak intensity; Cr is the core element for corrosion resistance, forming a dense chromium oxide film to isolate corrosive media; Ni strengthens the austenitic structure, enhancing material toughness and corrosion resistance; and Mn assists in regulating material structure, reducing costs, and improving machinability. Additionally, elements such as C, Ti, and V were detected. C participates in carbide formation, influencing grain structure; Ti fixes carbon, reducing intergranular carbide precipitation and enhancing resistance to intergranular corrosion; and V aids in grain refinement, strengthening the material. The overall elemental composition aligns with the alloy design of 316L stainless steel, where synergistic interactions among elements confer specific corrosion resistance and mechanical properties to the material.

#### 3.3.2. Response Surface Modeling

After completing calculations for 28 operating conditions, the settlement results were extracted and plotted as stress–strain curves. The four response values R_1_, R_2_, R_3_, and R_4_ were read from the curves and entered into the corresponding experimental design, as listed in the right column of Table 4. Software analysis yielded models describing the relationships between R_1_, R_2_, R_3_, and R_4_ and the damage model parameters. The *p*-value evaluation method was employed to assess the accuracy of the response surfaces. The *p*-values for all four parameters were significantly below the 0.05 significance level, indicating that these models are highly statistically significant. This confirms that the relationships between independent and dependent variables are not coincidental but statistically meaningful. All four values are very close to 1, indicating that the adjusted models fit the data well. Considering the number of independent variables, the models explain the dependent variables to a high degree. R^2^ is also close to 1, indicating that the independent variables in the model explain most of the variation in the dependent variables, and the model fit is high. The coefficient of variation (C.V.) values for the four response values are all less than 5%, indicating that the experimental results have good accuracy and reliability.

Analysis of the response surface model results (Table 5) indicates that the model significance level is <0.001, demonstrating statistical significance. The multiple correlation coefficient R^2^ > 0.95 indicates good data fitting. After evaluating the significance of each factor in the model, terms with insignificant effects on the response variable were removed from the regression model.

The resulting coded regression equation is as follows:

(3) $$\left\{ \begin{array}{l} R_{1}=0.157605-13.54691f_{0}-0.480479f_{n}+6.52877f_{c}+0.138894f_{f}\\ +125.93374f_{0}f_{n}+1070.29994f_{0}f_{c}+43.83021f_{0}f_{f}+79.41733f_{n}f_{c}+1.265f_{n}f_{f}\\ -18.21401f_{c}f_{f}-1526.76294{f_{0}}^{2}-95.85713{f_{n}}^{2}-310.04209{f_{c}}^{2}-0.152433{f_{f}}^{2}\\ R_{2}=712.03170-23738.18721f_{0}-664.48007f_{n}+1545.56094f_{c}+40.53485f_{f}\\ +1.12077\times 10^{5}f_{0}f_{n}+1.3892\times 10^{6}f_{0}f_{c}+44508.13802f_{0}f_{f}+43275.10127f_{n}f_{c}\\ +122.98177f_{n}f_{f}-14150.31829f_{c}f_{f}\\ R_{3}=0.241523-28.92085f_{0}-4.99837f_{n}+11.24283f_{c}+0.399174f_{f}\\ +1794.89063f_{0}f_{n}+1399.625f_{0}f_{c}+35.72063f_{0}f_{f}-583.3785f_{n}f_{c}-9.35516f_{n}f_{f}\\ -21.446f_{c}f_{f}-1795.23551{f_{0}}^{2}+196.54778{f_{n}}^{2}-103.96225{f_{c}}^{2}-0.98024{f_{f}}^{2}\\ R_{4}=641.642+15089.66816f_{0}+10259.34064f_{n}-9464.51758f_{c}-326.17615f_{f}\\ -4.34159\times 10^{6}f_{0}f_{n}+1.54041\times 10^{5}f_{0}f_{c}+15987.46875f_{0}f_{f}+1.2403\times 10^{5}f_{n}f_{c}\\ -10367.76719f_{n}f_{f}-29908.26806f_{c}f_{f} \end{array}\right.$$

#### 3.3.3. Effect of Damage Parameters on Material Tensile Curves

**Figure 16:**
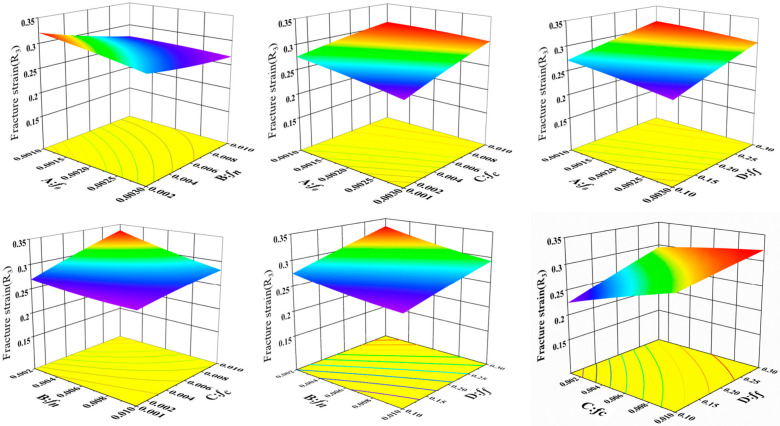
Response surface model for R3 interaction with damage parameters.

By comparing the coefficients of each factor in the coding equation, their relative impacts can be assessed, with high levels yielding positive values and low levels yielding negative values. The coding equation reveals that for R_1_, the absolute values of the *f*_0_ and *f_c_* coefficients are relatively large, indicating a significant influence on R_1_. Among these, the negative coefficient ${f_{0}}^{2}$ is the largest, exerting an inhibitory effect on R_1_. The positive coefficient *f*_0_*f_c_* is the largest, indicating its strongest positive driving effect on R_1_. For R_3_, the positive coefficient *f*_0_*f_n_* is the largest, indicating its strongest positive driving effect on R_3_. Among these, the negative coefficient ${f_{0}}^{2}$ is the largest, exhibiting an inhibitory effect on R_3_. Figure 16 presents the response surface model illustrating the interaction between the four parameters on the fracture point strain. Figure 16 reveals a strong interaction between *f_n_* and *f_c_*. As the formable nucleation pore volume fraction *f_n_* increases while the critical void volume fraction *f_c_* decreases, the fracture point strain of the material drops sharply.

**Figure 17:**
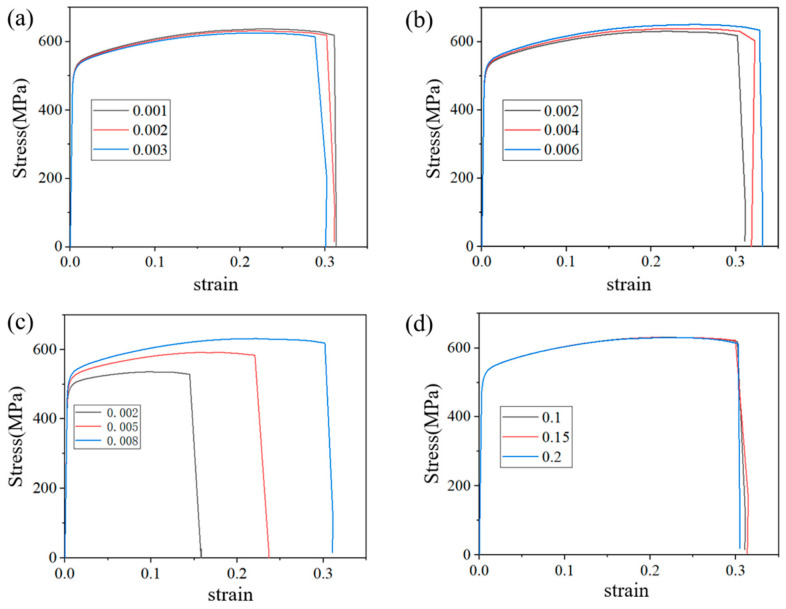
Effects of different parameters on tensile curves: (**a**) *f*_0_, (**b**) *f_n_*, (**c**) *f_c_*, (**d**) *f_f_*.

To investigate the influence of four damage parameters on the tensile stress–strain curve, simulations were conducted for three different initial pore fraction *f*_0_ conditions: 0.001, 0.002, and 0.003. The simulation results are shown in Figure 17a. As *f*_0_ increases from 0.001 to 0.003, the peak and fracture points of the tensile stress–strain curve slightly decrease. This occurs because as the initial pore volume fraction grows, the nucleation, coalescence, and growth of voids within the material during deformation advance, leading to material failure. For the nucleation pore volume fraction *f_n_*, simulations were conducted at three different parameter conditions: 0.002, 0.004, and 0.006. The simulation results are shown in Figure 17b. As the volume fraction of nucleation voids increases, both the peak and the fracture point of the curve shift slightly to the right. This is because, as the volume fraction of nucleation voids increases, the process by which the voids grow and reach the critical volume fraction takes longer, ultimately causing the fracture point and the peak to shift to the right. The critical pore volume fractions *f_c_* selected were 0.002, 0.005, and 0.008. As shown in the simulation results in Figure 17c, increasing the critical pore volume fraction causes significant changes in the stress–strain curve, with both the peak and fracture points increasing as the critical pore volume fraction grows. The fracture pore fraction was selected at three parameters: 0.1, 0.15, and 0.2. Simulation results are shown in Figure 17d. As the fracture pore fraction increases, the peak of the stress–strain curve remains unchanged, and the fracture point exhibits only a very small variation. This indicates that the fracture pore fraction has a limited overall effect on the curve.

#### 3.3.4. Optimal Parameter Calculation

This paper employs a constructed polynomial equation, using the measured tensile test response variable as the target value, and applies a genetic algorithm (GA) to iteratively solve for optimal parameters. Genetic algorithms are optimization search methods based on natural selection and genetic principles, achieving optimal solution searches by simulating the mechanisms of inheritance, mutation, and natural selection in biological evolution. Before algorithm execution, the initial population size was set to 50, the maximum number of evolutionary generations to 500, and the mutation probability to 0.9. Using data from Table 4 established by the response surface model in Section 3.3.2 as the processing object, the initial population parameters and maximum iteration count were reconfigured. The optimization variables are *f*_0_, *f_n_*, *f_c_*, and *f_f_.* Four characteristic parameters—the stress–strain values at the peak point and fracture point of the uniaxial tensile stress–strain curve at room temperature—are selected as optimization objectives. The parameter ranges are constrained to the intervals defined earlier.

**Figure 18:**
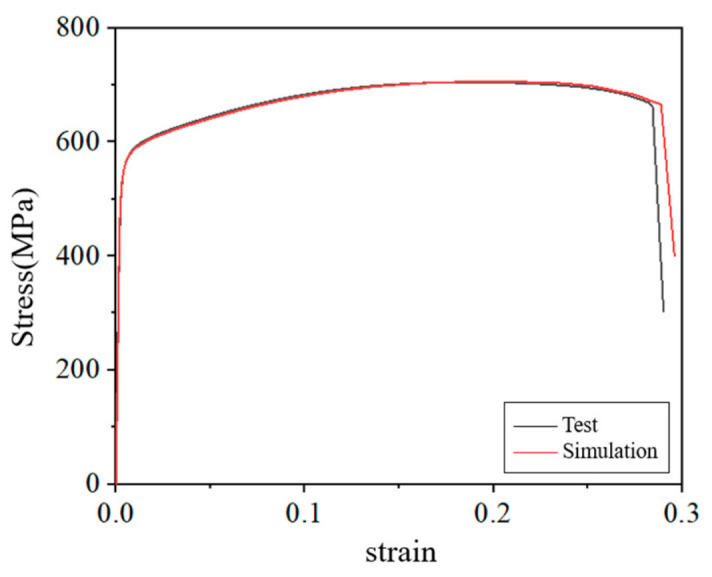
Comparison of stress–strain curves after parameter optimization.

The optimal parameters were obtained through calculation (Table 6) and compared with the corresponding test force–displacement curves (Figure 18). The simulation results showed good consistency with experimental data, with peak stress errors below 1% and fracture strain errors below 3%. This indicates that the GTN damage model optimized by the genetic algorithm can effectively predict progressive damage in SLM-manufactured 316L stainless steel. It should be noted that the inverse calibration of GTN parameters may suffer from non-uniqueness. The optimized parameters obtained in this study represent one physically constrained effective parameter set that reproduces the measured tensile response, rather than a mathematically unique solution.

#### 3.3.5. Model Validation

**Figure 19:**
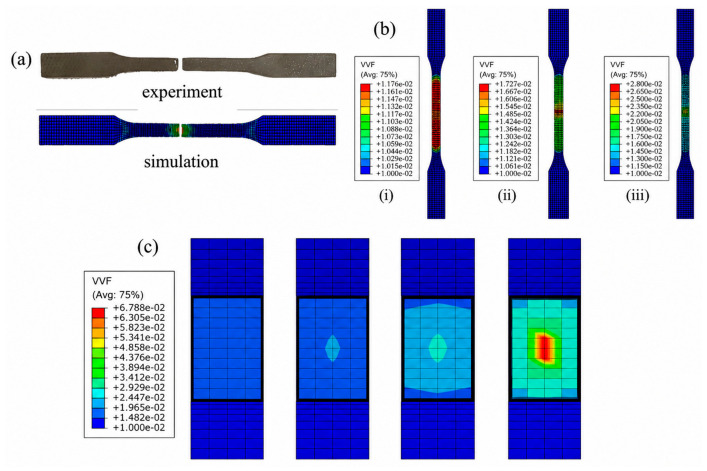
(**a**) Comparison chart of tensile specimen simulation tests; (**b**) VVF cloud diagram of tensile specimens at different displacements: (**i**) 5 mm; (**ii**) 10 mm; (**iii**) 11 mm; (**c**) VVF evolution at necking of uniaxial tensile simulation test.

Simulation calculations for tensile specimens were conducted using calibrated GTN model parameters. Figure 19a compares tensile test results with simulation outcomes, showing consistent fracture locations. This indicates the GTN damage model effectively predicts structural fracture damage, with damage parameters applicable to SLM-processed 316L tensile specimens. Figure 19 illustrates the evolution of VVF distribution during the compression fracture process of uniaxial tensile specimens.

Analysis based on specimen pore volume fraction (VVF) contour plots in Figure 19b shows that at a tensile displacement of 5 mm, the specimen undergoes uniform deformation with a VVF of 0.017, distributed uniformly across the tensile range and exhibiting low damage accumulation. As tensile displacement increased to 10 mm, pores began nucleating and rapidly growing within the specimen, expanding to 0.0172. This change resulted from deformation concentration effects triggered by pore aggregation. At a tensile displacement of 12.6 mm, pores not only continue nucleating but also exhibit rapid aggregation and growth, causing the pore volume fraction to surge sharply to 0.028 and further intensifying the deformation concentration trend. As shown in Figure 19c, the VVF contour map of the tensile specimen’s necking region clearly reveals that pores first aggregate in the necking area, with particularly pronounced clustering characteristics in the central region of the cross-section.

### 3.4. Influence of Defects on the Mechanical Properties of HLSHS

#### 3.4.1. Effect of Porosity

**Figure 20:**
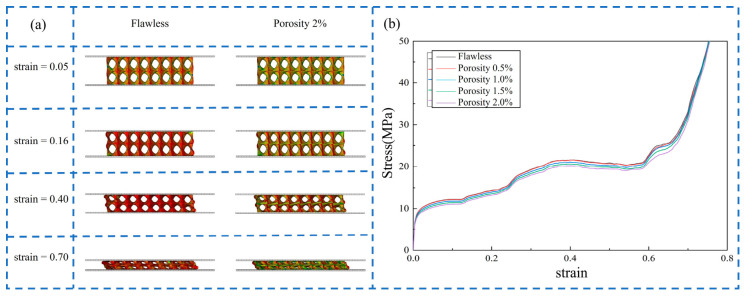
(**a**) Stress contour map of HLSHS at different porosities, (**b**) stress–strain curves of HLSHS at different porosities.

To investigate the influence of porosity on the mechanical properties of HLSHS, five different porosity parameters were set: no defect, 0.5%, 1%, 1.5%, and 2%. While other parameters remained constant, the stress–strain curves and energy absorption capacity during the compression deformation process were examined. Based on the GTN damage model parameters, damage parameters were recalibrated, and quasi-static compression simulations were conducted for HLSHS at different porosities. The figure below displays the stress contour plots and stress–strain curves during compression deformation for defect-free and 2% porosity structures. As shown in Figure 20a, the overall deformation process exhibits no significant differences. The primary distinction lies in the uneven stress distribution of the 2% porosity model, which is prone to stress concentration. Figure 20b indicates that during the initial elastic stage, simulation results show little variation across pore ratios. This is because internal porosity has minimal impact on material isotropy during elastic deformation. As the top plate continues compressing downward, it enters a plateau phase. At this point, increasing porosity reduces stress levels within the plateau region. At 0.5% porosity, the effect is limited due to the low pore content. However, as porosity further increases, stress levels begin to decrease significantly.

**Figure 21:**
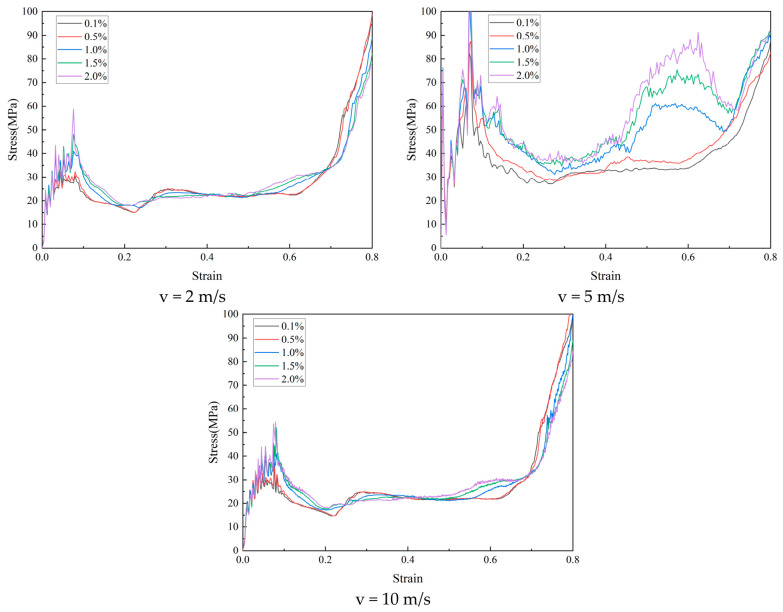
Dynamic response of HLSHS at different speeds.

The dynamic mechanical response of honeycomb structures is closely related to their porosity. By analyzing stress–strain data from honeycomb structures with varying porosities subjected to impact velocities of 2 m/s, 5 m/s, and 10 m/s (Figure 21), the influence mechanism of pore defects on their dynamic mechanical properties can be revealed.

As porosity increases, the peak stress of honeycomb structures exhibits an upward trend, leading to a decline in impact resistance. The presence of pores causes stress fluctuations within the structure during impact. At an impact velocity of 5 m/s, structures with low porosity (0.1%, 0.5%) exhibit a near-plateau phase following the peak impact force, characterized by minimal stress fluctuations and high energy absorption efficiency. In contrast, structures with high porosity (1.5%, 2%) display significant stress fluctuations during the plateau phase, which adversely affects their impact resistance.

#### 3.4.2. Effect of Wall Thickness Defects

**Figure 22:**
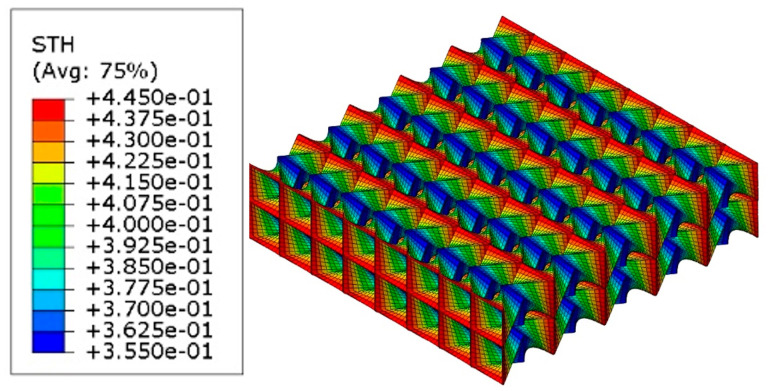
Schematic diagram of thickness distribution.

**Figure 23:**
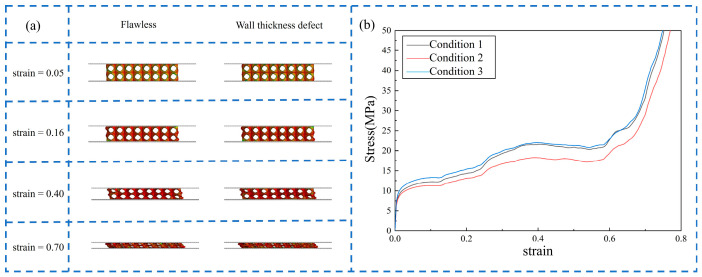
(**a**) Stress contour map of HLSHS with wall thickness defects, (**b**) stress–strain curves of HLSHS with different wall thickness defects.

To simulate the phenomenon of thickness non-uniformity occurring during additive manufacturing, a gradient thickness was applied to the finite element model of the HLSHS, as shown in Figure 22. Three operating conditions are set: Condition 1: Uniform thickness with no defects; Condition 2: Thickness gradient with overall thinning; Condition 3: Thickness gradient with overall mass unchanged. During compression, the deformation behavior of the thinned structure is largely consistent with that of the defect-free structure, differing only in stress levels. Figure 23b displays the stress–strain curves of HLSHS with varying thickness defects under quasi-static compression. Thickness defects have a limited impact on the elastic region but significantly affect the plateau region beyond it. Although stress levels decrease with reduced structural thickness, the overall mass remains constant despite the thickness gradient. Compared to defect-free structures, stress levels increase. This indicates that, beyond overall thickness, the thickness distribution also significantly influences the structural mechanical properties.

**Figure 24:**
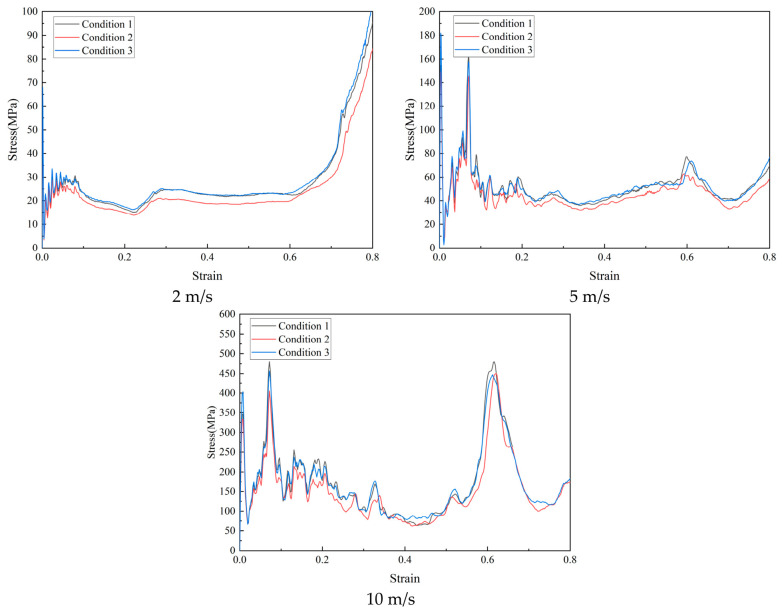
Dynamic response of HLSHS at different speeds.

The dynamic mechanical response of honeycomb structures is also closely related to their wall thickness. By analyzing stress–strain data from three honeycomb structures with different thickness distributions subjected to impact velocities of 2 m/s, 5 m/s, and 10 m/s (Figure 24), the influence mechanism of thickness defects on their dynamic mechanical properties can be revealed.

Substituting wall thickness defects into the dynamic impact simulation yields the results shown below. Specimens with progressively thinner walls exhibit reduced initial peak stresses. As the impact load persists, their subsequent stress values also decrease compared to standard specimens. Specimens with constant mass but uneven thickness distribution exhibit higher peak stresses than standard specimens during impact. However, under subsequent impact loading, their stress–strain curves become like those of standard specimens. Thinning the wall reduces energy absorption during impact, thereby diminishing the structure’s impact resistance. Uneven thickness distribution increases peak stresses during impact, also reducing the structure’s impact resistance.

## 4. Conclusions

This study fabricated HLSHS via selective laser melting (SLM). Internal defects were characterized using X-ray computed tomography (CT), defect evolution under loading was investigated via in situ tests, and finite element models were established to quantify the effects of porosity and wall thickness defects on mechanical performance. Key conclusions are as follows:

(1) HLSHS fabricated under the investigated high-quality process condition features low porosity, small and regular pores, and uniform wall thickness matching design specifications. Coarse process conditions lead to larger pore size, a sharp increase in irregular pores, and significantly amplified influence of pore characteristics on structural mechanical properties.
(2) Pores have a limited impact on tensile deformation and mechanical properties of specimens fabricated via high-quality additive manufacturing, with pore concentration/large pore regions decoupled from tensile fracture sites. In contrast, for specimens made under poor process conditions, pores dominate the deformation behavior: large pore regions highly coincide with fracture locations, and pore aggregation sites act as crack initiation points, resulting in an adverse effect on mechanical properties.
(3) Under compressive loading, pore defect evolution and wall thickness variation of the HLSHS mainly occur at structural corners. With increasing strain, the pore diameter expands from 0.71 mm to 2.54 mm under coarse process conditions and from 0.36 mm to 2.28 mm under fine process conditions. Meanwhile, the average wall thickness decreases from 0.568 mm to 0.53 mm under coarse process conditions and from 0.502 mm to 0.46 mm under fine process conditions.
(4) The increase in porosity reduces the quasi-static compressive stress of the structure, while simultaneously raising the stress peak under medium-to-high velocity impact and impairing its impact resistance. Wall thickness thinning similarly leads to a decrease in quasi-static compressive stress. Under the condition of constant total mass, an uneven wall thickness distribution results in higher structural stress compared to defect-free structures. Furthermore, an uneven wall thickness distribution also elevates the stress peak under medium-to-high velocity impact, adversely affecting impact resistance.

Future work will focus on constructing a broader process–defect–property database using a design-of-experiments approach. Additional defect descriptors, such as the pore aspect ratio, clustering, connectivity, and spatial distance from stress–concentration regions, will be incorporated into the model. Moreover, strain-rate-dependent constitutive models and dynamic experimental validation will be introduced to improve the predictive capability of impact simulations for SLM-fabricated HLSHS.

## Figures and Tables

**Table 1:** Density of 316L metal cubes fabricated by different processing techniques.

Test Number	Laser Power P (W)	Scanning Speed V (mm/s)	Scan Spacing S (mm)	Energy Density per Unit Volume (J/mm^3^)	Density (%)
T1	235	800	0.05	146.875	0.9974
T2	260	1000	0.05	130	0.9983
T3	285	1200	0.05	118.75	0.9978
T4	285	1000	0.1	71.25	0.9991
T5	260	800	0.1	81.25	0.9985
T6	235	1200	0.1	48.95833	0.9983
T7	235	1000	0.15	39.16667	0.9849
T8	260	1200	0.15	36.11111	0.9996
T9	285	800	0.15	59.375	0.9951

**Table 2:** Central composite design arrangement.

Level	*f*_0_	*f_n_*	*fc*	*f_f_*
−1	0.001	0.002	0.001	0.1
0	0.002	0.006	0.0055	0.2
1	0.003	0.01	0.01	0.3

**Table 3:** Damage parameter range.

Parameters	*f*_0_	*f_n_*	*fc*	*f_f_*
lower limit	0.001	0.002	0.001	0.1
upper limit	0.003	0.001	0.01	0.3

**Table 4:** Simulation results and corresponding response variables.

Group	Factor				Response Value			
	f_0_	f_n_	f_c_	f_f_	R_1_	R_2_	R_3_	R_4_
1	0.001	0.006	0.0055	0.2	0.180232	703.803	0.2923	565.088
2	0.003	0.002	0.001	0.3	0.179702	695.363	0.3154	584.612
3	0.002	0.01	0.0055	0.2	0.178805	701.988	0.2645	610.77
4	0.003	0.002	0.01	0.3	0.1818	701.472	0.31559	469.141
5	0.001	0.01	0.01	0.1	0.178759	703.368	0.24857	689.523
6	0.002	0.006	0.0055	0.2	0.180287	702.424	0.2861	594.612
7	0.002	0.002	0.0055	0.2	0.18175	702.853	0.3175	560.149
8	0.003	0.006	0.0055	0.2	0.180337	701.043	0.2761	591.364
9	0.002	0.006	0.01	0.2	0.180287	702.424	0.3041	553.329
10	0.001	0.002	0.01	0.1	0.181698	704.236	0.32114	534.763
11	0.003	0.002	0.001	0.1	0.128268	652.429	0.1676	638.637
12	0.002	0.006	0.0055	0.2	0.180287	702.424	0.2861	594.612
13	0.001	0.01	0.001	0.1	0.154281	693.822	0.1979	671.693
14	0.001	0.01	0.001	0.3	0.1747	701.543	0.3005	594.537
15	0.003	0.01	0.01	0.1	0.178728	704.336	0.2525	679.703
16	0.002	0.006	0.0055	0.2	0.180287	702.424	0.2861	594.612
17	0.001	0.002	0.001	0.1	0.170127	699.647	0.2278	644.835
18	0.001	0.002	0.001	0.3	0.180339	703.535	0.3416	528.407
19	0.002	0.006	0.0055	0.1	0.180287	702.424	0.2421	633.617
20	0.003	0.01	0.001	0.3	0.171942	693.512	0.2876	546.125
21	0.003	0.01	0.001	0.1	0.122742	649.899	0.16	607.894
22	0.002	0.006	0.0055	0.2	0.180287	702.424	0.2861	594.612
23	0.001	0.002	0.01	0.3	0.181698	704.236	0.3406	447.244
24	0.002	0.006	0.001	0.2	0.170779	693.736	0.2545	608.74
25	0.003	0.002	0.01	0.1	0.1818	701.472	0.2891	595.041
26	0.001	0.01	0.01	0.3	0.178759	703.368	0.3314	540.788
27	0.002	0.006	0.0055	0.3	0.180287	702.424	0.3407	530.087
28	0.003	0.01	0.01	0.3	0.178853	700.61	0.3126	517.138

**Table 5:** Regression analysis results.

	R_1_	R_2_	R_3_	R_4_
*p*-value	<0.0001	<0.0001	<0.0001	<0.0001
$R_{adj}^{2}$	0.9781	0.985	0.9823	0.9811
R2	0.9719	0.9789	0.9804	0.988
C.V.%	4.39	0.2539	1.32	0.8402

**Table 6:** Optimized GTN damage parameters.

Parameters	*f*_0_	*f_n_*	*fc*	*f_f_*
Value	0.00251	0.00804	0.009	0.13

## Data Availability

The original contributions presented in this study are included in the article. Further inquiries can be directed to the corresponding author.
